# Important Dependency-Associated Community Resources among Elderly Individuals with a Low Level of Social Support in China

**DOI:** 10.3390/ijerph18052754

**Published:** 2021-03-09

**Authors:** Ying Li, Yiyang Pan, Yuan Chen, Pingyu Cui

**Affiliations:** Department of Social Medicine, School of Public Health, Zhejiang University, Hangzhou 310058, China; 54192670@zju.edu.cn (Y.P.); 68188247@zju.edu.cn (Y.C.); shiyu516@hotmail.com (P.C.)

**Keywords:** dependency, community resources, low level of social support, elderly

## Abstract

*Background:* The prevalence of dependency personality disorder is high among elderly individuals with a low level of social support. The objective of this study was to explore the dependency associated with important community resources among elderly individuals with a low level of social support from the perspective of resource demand. *Methods:* The population-based cross-sectional study was conducted in 22 locations in China. A total of 950 participants aged ≥60 years were selected using a complex multistage sampling design. All the data were collected using questionnaires via face-to-face interviews. The dependency was assessed using the standardized Chinese version of the Minnesota Multiphasic Personality Inventory-II. Community resources were assessed using 43 items. Logistic regression analysis was used to evaluate the association between dependency and important community resources. *Results:* Bivariate analysis showed that the level of social support was negatively associated with levels of income (*p* < 0.001) and education (*p* = 0.008) and was positively associated with social communication and interactions (*p* < 0.001). The logistic regression analysis showed that the emergency call or survival monitoring system (ECSMS) was the most important community resource that was significantly associated with the levels of dependency; the odds ratio was 2.64 (95% CI, 1.07–3.91; *p* = 0.031) among elderly individuals with a low level of social support. *Conclusions:* The levels of dependency were most significantly associated with the ECSMS among elderly individuals with a low level of social support. Our results suggest that improving the ECSMS can be the main problem in the development of community resources.

## 1. Introduction

With the rapidly increasing aging population, solving the allocation of community health service resources that meet the needs of the elderly is important, especially for individuals with poor social support and low income [1,2]. The increasing prevalence of their dependency leads to increasing demand for medical and social services [3]. Although dependency has become an urgent problem requiring immediate solutions, a recent study has shown that the relationship between dependency and health status or the provision of support is complicated and by no means a simple one [4].

Traditional dependency is a personality disorder in which individuals are highly dependent on others to fulfill their emotional and physical needs, resulting in the gradual loss of autonomy [5,6]. Dependency can lead to disability and premature functional dependency and is associated with longer hospital stays, increased healthcare costs, and adverse effects on the physical and mental health of caregivers [7,8]. Epidemiological studies have shown that dependency is associated with depression, suicide, and all-cause mortality, and leads to increases in consequences of ill health [9,10]. A study demonstrated a rate of transition to dependency in a high proportion of patients beyond the rehabilitation provided. All patients have a greater need for health surveillance and community support after hospital discharge [11].

In the past, the main focus of dependency studies was mostly on the negative consequences. Recently, studies have shown that dependency on health services agencies can be described as a positive condition for patients because it helps them feel as if they are in a safe and comfortable place [12]. The theory of resource dependency emphasizes that the survival of an organization needs to absorb resources from the surrounding environment and needs to depend on and interact with the surrounding environment to achieve better survival [13,14]. Some studies have shown that place dependency is positively associated with the service quality of the place [15]. In other words, the better the service of a place, the more dependent the individual is on the place. More recently, dependency on community services has been considered as being on a continuum of more or less autonomy, and the situation might be an often necessary and helpful temporary phase toward autonomy. It is the transitory and ever-changing state of dependency on community services that makes dependency not an entirely negative phenomenon [16].

With aging, elderly individuals become more vulnerable to environmental challenges [17]. The community environment is particularly important for the well-being of elderly individuals, especially for their mental health [18]. A longitudinal study has identified the availability of social resources as an important factor in the likelihood of assistance with dependency. Moreover, resource inequality has been identified as a barrier to covering the necessary long-term support for independency [19].

The World Health Organization suggests promoting aging-in-place (AIP) policies for both developed and developing countries [20]. AIP refers to “the ability to live in their own home and community safely, independently, and comfortably, regardless of age, income, or ability level”. It contributes to the well-being of elderly individuals by providing a sense of attachment, connectedness, security and familiarity, identity, autonomy, and independence [21,22,23,24]. Although it is recognized that elderly individuals generally prefer to stay in their homes and communities for as long as possible, the situation is more complicated for elderly individuals with low income who lack financial resources and care support for their preferred mode of living when faced with physical decline. However, more than 80% of elderly individuals expressed a wish to remain living in the community even when their health and functioning deteriorated to the point where they could no longer live alone. With the increasing number of elderly living in the community, various problems have been raised, which bring new challenges to community medical services. At present, many more advanced apartments and modern elderly community for the elderly have been constructed in China. However, most of them are elderly with low income and level of social support, the community health services and facilities that they live in are imperfect, and many communities do not even have an emergency service system.

The main objective of this study was to assess the community resources associated with dependency among elderly individuals with a low level of social support. On the basis of the theory of resource dependency and AIP policies, we want to know the community resources that the elderly are dependent. We hypothesized that the elderly can live in their familiar communities for a longer time by providing or improving these resources.

## 2. Materials and Methods

### 2.1. Subjects

This study was based on the “Accessibility Evaluation of Health-Related Resources for the Elderly” project using a cross-sectional design. A total of 950 community residents aged ≥60 years were selected using a multistage sampling design. Sampling was conducted in 22 locations in four provinces (Zhejiang, Heilongjiang, Xinjiang, and Sichuan) in China during the period from July 2019 to November 2019. A population-based survey was conducted to assess the health and health-related resources of accessibility of the elderly population from the following aspects: socioeconomic, behavioral and psychological, medical, and environmental.

Study excluded subjects who were unable to complete the questionnaire. All participants provided written informed consent before participation in this study. The study was approved by the institutional review board at the School of Medicine, Zhejiang University (ZGL201907-10).

### 2.2. Data Collection

Data were collected through face-to-face interviews using structured questionnaires. The questionnaire is divided into 9 parts and comprises 428 items. The main content comprised demographics, general health status, behavioral habits, environmental and community health service resources, socioeconomic resources, psychological resources, and activities of daily living assessment. Before the formal investigation, we produced a preliminary survey to verify the validity of the questionnaire. The duration of the interview was approximately 45 to 60 min for the majority of participants. Many of the elderly needed more time; some of them even asked to stop taking part in the investigation. The interviewer suggested that they may take a rest and asked if they would like to complete the questionnaire. Those who did not agree to continue were excluded from the study.

The general characteristics of the participants included the following: age, gender, ethnicity, income, education level, self-reported chronic disease status, daily habits, and physical activity level. Environmental assessment is generally conducted from three aspects: physical, social, and psychological. In this study, community environmental resources were assessed using 43 items. The main questions are as follows: “Does your community have an emergency call or survival monitoring system (ECSMS) or provide corresponding services?” “Do you think it is necessary for someone to check and assess your overall health in this way?” “Does your community hold regular lectures on health knowledge?” If respondents responded with “yes”, then the answer was coded as “1”; otherwise, it was coded as “0”. For the question “In the past six months, how many times have you been to community health service organizations for treatment or received a general practitioner’s on-site service?”, the answers were divided into four levels including “<2 times”, “2–3 times”, “4–6 times” and “>6 times”. Income satisfaction was measured by the following question: “Do you think your income is enough to meet your general needs?” The responses were recorded using four levels as follows: “sufficient”, “just enough”, “slightly inadequate” and “wholly inadequate”.

The Chinese version of the dependency scale used in this study was validated by the standardized Minnesota Multiphasic Personality Inventory-II [25]. The dependency scale comprised 57 items. The raw score was calculated and converted into a standardized T-score. A score of 60 or above indicate meeting the criteria for dependency personality disorder. Social resource status was assessed using the Chinese version of the questionnaires of the Older American Resources and Services (OARS) social resource scale. The ratings were summed to yield a total score. A high level of social support was defined as an OARS score greater than or equal to 11 points. Participants’ personality characteristics were measured using the Eysenck Personality Questionnaire (EPQ).

### 2.3. Statistical Analysis

The present analysis was conducted with 913 participants who had complete questionnaires and dependency assessment data. The characteristics of the study participants were described using descriptive statistics analysis. We calculated percentages for categorical variables and calculated the means and standard deviations for the continuous variables.

The chi-squared test was used to perform bivariate analysis for the level of social support and related factors. For data that did not meet the requirement of the chi-squared test, Fisher’s exact test was used. The participants were divided into two groups, high level of social support and low level of social support, by OARS scores. All statistical tests used a two-sided test, and a value of *p* < 0.05 indicated statistical significance.

We used a separate logistic regression model to evaluate the dependency-associated community resources between the elderly with low and high levels of social support. If the participant’s T-score on the dependency scale was greater than or equal to 60 points, then they were regarded as dependent individuals in the binary dependent variable of the logistic regression model, expressed by “1,” and “0” was used for scores lower than 60 points. A total of 43 community resource variables with a value of *p* < 0.05 in the univariate analysis were included in the multivariate logistic regression analysis. In the model, dependency was the outcome variable, community resources were the predictive variable, and the covariates included age, gender, income satisfaction, alcohol use, smoking status, physical activity, chronic disease status, Geriatric Depression Scale (GDS-15) scores, and EPQ scores. All analyses were performed using SAS for Windows (version 9.4).

## 3. Results

The characteristics of the study participants by gender are shown in Table 1. Among all the participants, 533 (58.4%) were female, and 380 (41.6%) were male. Approximately 22.3% lived in cities, and 77.7% lived in town and rural areas. A total of 66.7% of participants self-reported that they had one or more chronic diseases. The average dependency score showed no significant difference between males and females (*p* = 0.664).

The analysis results of the level of social support and associated risk factors in the chi-squared test are presented in Table 2. In the group aged 70 years and over, 65.7% of participants had a low level of social support (*p* = 0.004). The level of social support was negatively associated with education level (*p* = 0.008) and individual income (*p* < 0.001). Elderly people living alone have a greater likelihood of a low level of social support. Both the frequency of chatting with mobile phones and times visiting friends or going out in the past week were positively associated with a high level of social support (*p* < 0.001). In the low-level social support group, the proportion of service utilization for establishing health records and family doctor contract services was significantly higher than that in the high-level social support group. No significant difference was found in community-provided services for the ECSMS between the two groups with high and low levels of social support.

The results of the dependency association with community resources by logistic regression analysis are shown in Table 3. In the group with a low level of social support, the ECSMS was most significantly associated with the levels of dependency: the OR was 2.64 (95% CI, 1.07–3.91; *p* = 0.031). Other community environmental resources, such as the need for health assessment and regular lectures on health knowledge, were significantly positively associated with levels of dependency with ORs of 2.04 (95% CI, 1.06–3.94; *p* = 0.033) and 1.82 (95% CI, 1.13–2.93; *p* = 0.014), respectively. Both libraries and cafes or tea rooms were negatively associated with levels of dependency with ORs of 0.39 (95% CI, 0.19–0.80; *p* = 0.010) and 0.35 (95% CI, 0.18–0.67; *p* = 0.001), respectively.

The group with a high level of social support was different from the group with a low level of social support, and a community ECSMS was not significantly associated with the levels of dependency. Received community health services and community geriatric wards were significantly associated with levels of dependency with ORs of 1.35 (95% CI, 1.09–1.67; *p* = 0.006) and 2.37 (95% CI, 1.31–4.27; *p =* 0.004), respectively. As the group with a high level of social support, the need for health assessment was also associated with the level of dependency with an OR of 2.03 (95% CI, 1.44–6.37; *p* = 0.004).

## 4. Discussion

In this study, we found that dependency was significantly associated with community resource use. We focused more on elderly individuals with a low level of social support. The present results suggest that the ECSMS is the most important community resource for elderly individuals with a low level of social support, and is associated with dependency. The association was not observed in elderly individuals with high levels of social support.

In recent years, the prevalence of dependency has continuously increased among community elderly individuals due to population aging, but this is only part of a variety of complex reasons. Another equally important aspect is the change in social and economic landscapes [26,27,28]. In some traditional and low-income countries, elderly individuals are living with close relatives and always receive care from their children or other family members when they suffer from diseases or disability [29,30]. With changes in population structures and socioeconomic status, elderly individuals have lost their traditional unpaid care resources from family support. In addition, a recent study has shown that the number of care homes has decreased over the past two decades, and an increasing number of elderly people choose to live in their own homes [31]. The common characteristics of elderly individuals are that they do not live with their families, have fewer social interactions, have a sense of loneliness, and are often left unattended when ill [32]. In the present study, elderly individuals with a low level of social support were also characterized by a low level of education and income and had a significantly lower frequency of mobile chatting or visiting friends than elderly individuals with a high level of social support. Along with the growth of age, elderly individuals become increasingly more lonely, insecure, and more vulnerable to environmental challenges, but there is relatively little information on statutory community services [33]. The main reason for the result was that dependency is probably widely underrecognized and receives an inadequate clear call to act after it is recognized. A study suggests that an evidence-based approach is necessary to understand the interactions between community resources and dependency among the elderly population, but there are few studies conducting a comprehensive analysis of elderly individuals with a low level of social support [34]. We calculated the average score of dependency for different regions. Compared with the urban elderly, the rural elderly had a higher average score of dependency. However, the region is a covariate placed in the two separate logistic regression models that were not observed to be associated with dependency (the results did not show) [35]. To our knowledge, quantitative analysis is rarely used to assess the association between dependency and community health resources.

A few previous studies have discussed the association between dependency among elderly individuals with a low level of social support and community resources from the perspective of resource dependency [36,37]. The understanding of dependency on community services usually has negative characteristics, such as dependency, which is significantly associated with the direct medical, social, informal, and total care costs and adverse physical and mental health outcomes for caregivers [38,39]. However, a study suggested that dependency was not a totally negative or positive concept [40]. Dependency on health services can be described as a positive condition for patients because it helps them feel as if they are in a safe and comfortable place. Our study found that the ECSMS is the most important community resource and was associated with dependency among elderly individuals with a low level of social support. The results reveal that most elderly individuals with a low level of social support and dependency tend to live in communities with an ECSMS. Our results are similar to those of a recent study in which low-income elderly individuals prefer to age in place, and perceived proximity to medical facilities is a crucial factor [41]. Based on the theory of resource dependency, we may explain the phenomenon as follows: elderly individuals with a low level of social support depend on the ECSMS to obtain a sense of security through perception. In this study, the elderly live in Hong Kong, one of the most developed cities in China. Different from the elderly in our study, most of whom have their own housing, the many of the elderly in Hong Kong live in subsidized public rental housing. However, the elderly living in subsidized public rental housing also like to live in a community. Hong Kong has a comprehensive long-term care system that provides for the elderly, which, however, does not seem to be the main reason for the elderly choose to live in the community. The study showed the unique role of medical facilities among community facilities in a modernized Chinese city. This result is similar to ours, and libraries and cafe or tea rooms are important facilities that promote social connectedness and feeling less alone.

Dependency in the present study may be described as a positive condition for elderly people with a low level of social support because it helps them feel as if they live in a safe place. From the perspective of community services, we can establish and improve ECSMSs to meet the resource needs of elderly individuals with low-level social support to reduce dependency and increase independence. Furthermore, only the need for health assessment was associated with two groups: a low level of social support and a high level of social support. We further observed that dependency was positively associated with receiving community health services and community geriatric ward services among elderly individuals with a high level of social support. The current result is similar to a previous study in which elderly individuals with a high level of social support were more dependent on high-payment community health services and tended to have increased healthcare costs and hospital stays [42]. In this study, compared with elderly individuals with a high level of social support, dependency was mainly associated with a free regular lecture on health knowledge among elderly individuals with a low level of social support. In addition, elderly people with a low level of social support have a high willingness to establish health records and family doctor contract services, but they cannot obtain substantive health services. These results revealed that the available community health resources are unequal in the elderly population between different levels of social support groups. Elderly people in low-level social support groups are more likely to use free and easily accessible community health resources.

Our research suggests that the ECSMS is the most important community resource need of elderly people with a low social support level. The construction of a community ECSMS can enhance the sense of connectedness and security for elderly people with a low social support level. However, an ECSMS is a community service resource that needs prior consideration and a solution for elderly individuals with low levels of social support because it can effectively reduce their sense of insecurity and helplessness and enhance their autonomy; however, dependency is a very complex problem that cannot be solved in a simple way. We can consider intervention strategies comprehensively based on some findings of our study, such as increasing and improving community service resources such as tea rooms and libraries to provide more opportunities for social interaction for elderly individuals, reduce loneliness, increase the use of these service resources, reduce the use of medical service resources, increase autonomy and reduce dependency. Furthermore, from the perspective of health equity, we should enact feasible strategies to encourage elderly individuals with low incomes to use general community health services, to instill a sense of identity and familiarity in them, and to help them live longer in homes and communities they do not want to leave.

The main strength of this study is that we collected full data to enable us to consider more factors for the analysis and improve the reliability of the study results. We used a quantitative analysis method to help us evaluate the associated strength between the ECSMS and dependency for elderly individuals with a low level of social support. In addition, unlike some previous studies, the present study controlled for a large number of potential risk factors, including personality factors and depression. This study also has several limitations. First, it is a cross-sectional study. We can verify the associations between dependency, the ECSMS and other factors but cannot determine causality. Second, the cause and mechanism of dependency are complex. We are interested in further exploring the interaction between different factors, such as social, environmental and psychological factors, but we could not obtain a sufficiently large sample size to evaluate the interactions. Third, a potential reporting bias exists in this study. The community resources are self-reported; they should not exclude individuals with higher dependency and, as a personality trait, are more aware of the resources available to them in the community. Fourth, the participants were not given the option to answer “I don’t know” with regards to community resources; therefore, the participants are more likely to answer “No,” which can lead to reporting bias. In addition, we defined elderly people as ≥60 years old according to the law of the People’s Republic of China on the Protection of the Rights and Interests of the Elderly. However, in many developed countries, elderly people are usually defined as ≥65 years old. The World Health Organization calls 60–74 years old as young elderly, 75–89 years old as ordinary elderly, and over 90 years old as the longevous elderly. The health conditions and needs of the elderly in different age groups are rather different. Therefore, the present result extrapolation is difficult. However, further qualitative study is required to clarify the mechanism underlying this association and to advance our knowledge of the role of an ECSMS among elderly individuals.

## 5. Conclusions

In this study, we explored the dependency associated with community resources among elderly individuals with a low level of social support from the perspective of resource demand. Our results showed that the ECSMS is the most important community resource associated with dependency for elderly individuals with a low level of social support. We suggest that elderly individuals with a low level of social support often lack available social contacts and communication and are accompanied by low income and unattainable community medical resources. Compared with elderly individuals with a high level of social support, elderly individuals with a low level of social support lack a sense of security. Therefore, the establishment and improvement of community ECSMSs is an urgent and key action to help these elderly individuals live in their familiar communities safely, automatically, and independently. However, it is very challenging work to solve the problem of elderly individuals with dependency. For elderly individuals with a low level of social support, we should not only give priority to solving the ECSMS, but we should also take further effective intervention strategies to promote the social interaction of these elderly individuals and provide accessibility to community medical resources. However, further research is needed to reduce the health divide caused by the level of social support to allow elderly individuals with a low level of social support to live in their familiar community independently and happily.

## Figures and Tables

**Table 1:** Characteristics of study participants in the study.

Variable Categories	Men (*N* = 380)		Women (*N* = 533)	
	*n*	%	*n*	%
Age (year)				
60–69	228	(60.0)	341	(64.0)
≥70	152	(40.0)	192	(36.0)
Region				
City	60	(15.8)	144	(27.0)
Town	200	(52.6)	277	(52.0)
Rural	120	(31.6)	122	(21.0)
Education (year)				
0–6	44	(11.6)	132	(24.8)
7–9	163	(42.9)	190	(35.6)
10–12	96	(25.3)	115	(21.6)
13+	77	(20.2)	96	(18.0)
Individual income				
¥0 to 1999	232	(61.1)	318	(59.7)
¥2000 to 3999	80	(21.1)	150	(28.1)
¥4000 and Over	68	(17.8)	65	(12.2)
Smoking status				
Yes	118	(31.1)	8	(1.5)
No	262	(68.9)	525	(98.5)
Alcohol use				
Yes	146	(38.4)	40	(7.5)
No	234	(61.6)	493	(92.5)
Physical activity				
Yes	130	(34.2)	230	(43.2)
No	250	(65.8)	303	(56.8)
Chronic disease status				
Yes	259	(68.2)	352	(66.0)
No	121	(31.8)	181	(34.0)
Income satisfaction				
Sufficient	87	(22.8)	118	(22.2)
Just enough	148	(38.9)	212	(39.9)
Slightly inadequate	82	(21.5)	122	(22.9)
Wholly inadequate	64	(16.8)	80	(15.0)
Measured Variables (Mean, SD)				
Dependency scores	43.8	(11.6)	43.2	(13.7)
Depression scores	3.2	(2.9)	3.6	(3.2)
EPQ scores	21.9	(3.8)	22.7	(4.1)

EPQ: Eysenck Personality Questionnaire.

**Table 2:** The level of social support for characteristics of participants in Chi-square test.

Variable	Low Level of Social Support		High Level of Social Support		*p* Value
	*n*%		*n*%		
Age (year)					
60–69	320	(56.2)	249	(43.8)	0.004
≥70	226	(65.7)	118	(34.3)	
Education (year)					
0–6	122	(69.3)	54	(30.7)	0.008
7–9	215	(60.9)	138	(39.1)	
10–12	117	(55.5)	94	(44.5)	
13+	92	(53.2)	81	(46.8)	
Individual income					
¥0 to 1999	354	(64.4)	196	(35.6)	<0.001
¥2000 to 3999	128	(55.7)	102	(44.3)	
¥4000 and Over	64	(48.1)	69	(51.9)	
Living arrangement					
Living alone	58	(95.1)	3	(4.9)	<0.001
Living with spouse	234	(52.8)	209	(47.2)	
Living with children	165	(63.7)	94	(36.3)	
Others	89	(59.3)	61	(40.7)	
Mobile chat (times/past week)					
0	142	(91.6)	13	(8.4)	<0.001
1	211	(82.4)	45	(17.6)	
≥2	193	(38.4)	309	(61.6)	
Visiting friends or out (times/past week)					
0	322	(90.4)	34	(9.6)	<0.001
1	160	(62.3)	97	(37.7)	
≥2	64	(21.3)	236	(78.7)	
Establishing health records					
Yes	364	(63.2)	212	(36.8)	0.006
No	182	(54.0)	155	(46.0)	
Family doctor contract service					
Yes	305	(66.2)	156	(33.8)	<0.001
No	241	(53.3)	211	(46.7)	
Emergency call or survival monitoring system					
Yes	65	(47.8)	71	(52.2)	0.223
No	328	(42.2)	449	(57.8)	

**Table 3:** The odds ratios of dependency association with community resource that are presented separately for low social support and high social support.

Variables	Multivariable Adjusted			*p* Value
	Odd Ratios	95% CI		
Low level of social support				
Emergency call or survival monitoring system (no/yes)	2.64	1.07	3.91	0.031
The need for health assessment (no/yes)	2.04	1.06	3.94	0.033
Regular lectures on health knowledge (no/yes)	1.82	1.13	2.93	0.014
Library (no/yes)	0.39	0.19	0.80	0.010
Cafe or tea room (no/yes)	0.35	0.18	0.67	0.001
Chronic disease status (no/yes)	1.71	1.02	2.87	0.040
EPQ scores (points)	1.13	1.07	1.19	<0.001
GDS-15 scores (points)	1.50	1.28	1.64	<0.001
Age (year)	1.01	0.97	1.05	0.762
Gender	0.96	0.50	1.85	0.905
Alcohol (no/yes)	1.16	0.78	1.85	0.464
Smoking status (no/yes)	1.13	0.73	1.74	0.598
Physical activity (no/yes)	0.87	0.43	1.80	0.723
Income satisfaction degree	1.13	0.86	1.49	0.384
High level of social support				
Emergency call or survival monitoring system (no/yes)	0.46	0.16	1.30	0.145
Received community health services (times)	1.35	1.09	1.67	0.006
The need for health assessment (no/yes)	2.03	1.44	6.37	0.004
Community geriatric ward (no/yes)	2.37	1.31	4.27	0.004
Income satisfaction degree	1.65	1.34	2.03	<0.001
GDS-15 scores (points)	1.54	1.41	1.68	<0.001
Age (year)	1.08	0.99	1.07	0.147
Gender	1.09	0.64	1.86	0.731
Alcohol (no/yes)	0.75	0.55	1.03	0.075
Smoking status (no/yes)	1.08	0.79	1.48	0.624
Physical activity (no/yes)	1.12	0.65	1.93	0.695
Chronic disease status (no/yes)	1.25	0.80	1.96	0.322

GDS: Geriatric Depression Scale.

## Data Availability

The datasets used and/or analyzed during the current study are available from the corresponding author on reasonable request.

## References

[1] Beard J.R., Officer A., de Carvalho I.A., Sadana R., Pot A.M., Michel J.P., Lloyd-Sherlock P., Epping-Jordan J.E., Peeters G.M.E.E.G., Mahanani W.R. (2016). The World report on ageing and health: A policy framework for healthy ageing. Lancet.

[2] Chang A.Y., Skirbekk V.F., Tyrovolas S., Kassebaum N.J., Dieleman J.L. (2019). Measuring population ageing: An analysis of the Global Burden of Disease Study 2017. Lancet Public Health.

[3] World Health Organization (2015). World Report on Ageing and Health.

[4] Feng Z.L. (2019). Global Convergence: Aging and Long-Term Care Policy Challenges in the Developing World. J. Aging Soc. Policy.

[5] Disney K.L. (2013). Dependent personality disorder: A critical review. Clin. Psychol. Rev..

[6] Friedman A.F., Lewak R., Nichols D.S., Webb J.T. (2001). Psychological assessment with the MMPI-2. Appendix A: Scoring keys and norms for MMPI-2 scales and critical items. Psychological Assessment with the MMPI-2.

[7] Perales N.M.G., Jiménez B.R., Caballero D.C., González B.M., Juarez L.M. (2020). Informal Cares and Caregivers in Rural Elderly: Emotional Costs in Public Health Policies. Handbook of Research on Health Systems and Organizations for an Aging Society.

[8] Silva A.L., Teixeira H.J., Teixeira M.J.C., Freitas S. (2013). The needs of informal caregivers of elderly people living at home: An integrative review. Scand. J. Caring Sci..

[9] Lahortiga-Ramos F., Unzueta C.R., Zazpe I., Santiago S., Molero P., Sánchez-Villegas A., Martínez-González M.Á. (2018). Self-perceived level of competitiveness, tension and dependency and depression risk in the SUN cohort. BMC Psychiatry.

[10] Bornstein R.F. (2012). Illuminating a neglected clinical issue: Societal costs of interpersonal dependency and dependent personality disorder. J. Clin. Psychol..

[11] Queralt-Tomas L., Clua-Espuny J.L., Fernández-Saez J., Lleixà-Fortuño M.M., Albiol-Zaragoza I., Gil-Guillen V., Carratala-Munuera C. (2019). Risk of Dependency: A Challenge for Health and Social Care Planning-Observational Stroke Cohort. Value Health..

[12] Lengen C., Timm C., Kistemann T. (2019). safe and comfortable place and life path trajectories: The development of a place-time-identity model. Soc. Sci. Med..

[13] Yeager V.A., Zhang Y.K., Diana M.L. (2015). Analyzing Determinants of Hospitals’ Accountable Care Organizations Participation: A Resource Dependency Theory Perspective. Med. Care Res. Rev..

[14] Yeager V.A., Menachemi N., Savage G.T., Ginter P.M., Sen B.P., Beitsch L.M. (2014). Using resource dependency theory to measure the environment in health care organizational studies: A systematic review of the literature. Health Care Manag. Rev..

[15] Yu M., Kelley A.T., Morgan A.U., Duong A., Mahajan A., Gipson J.D. (2020). Challenges for Adult Undocumented Immigrants in Accessing Primary Care: A Qualitative Study of Health Care Workers in Los Angeles County. Health Equity.

[16] Bonavigo T., Sandhu S., Pascolo-Fabrici E., Priebe S. (2016). What does dependency on community mental health services mean? A conceptual review with a systematic search. Soc. Psychiatry Psychiatr. Epidemiol..

[17] Walsh J.J. (2020). Meeting compliance under Medicare and Medicaid emergency preparedness Final Rule requirements. J. Bus. Contin. Emer. Plan..

[18] Jopp D.S., Boerner K., Ribeiro O., Rott C. (2016). Life at Age 100: An International Research Agenda for Centenarian Studies. J. Aging Soc. Policy.

[19] Pinfold V. (2000). ‘Building up safe havens… all around the world’: Users’ experiences of living in the community with mental health problems. Health Place.

[20] World Health Organization (2007). Global Age-Friendly Cities: A Guide.

[21] Jeste D.V., Blazer D.G., Buckwalter K.C., Cassidy K.K., Fishman L., Gwyther L.P., Levin S.M., Phillipson C., Rao R.R., Schmeding E. (2016). Age-Friendly Communities Initiative: Public Health Approach to Promoting Successful Aging. Am. J. Geriatr. Psychiatry.

[22] Baron M., Fletcher C., Riva M. (2020). Aging, Health and Place from the Perspective of Elders in an Inuit Community. J. Cross Cult. Gerontol..

[23] Vanleerberghe P., De Witte N., Claes C., Schalock R.L., Verté D. (2017). The quality of life of older people aging in place: A literature review. Qual. Life Res..

[24] Steptoe A., Deaton A., Stone A.A. (2015). Subjective wellbeing, health, and ageing. Lancet.

[25] (2004). Minnisuda Dou Xiang Ren Ge Ce Yan Zhong Wen Ban Yong Hu Shou Ce.

[26] Lee J., Phillips D., Wilkens J., Chien S., Lin Y.C., Angrisani M., Crimmins E. (2018). Cross-Country Comparisons of Disability and Morbidity: Evidence from the Gateway to Global Aging Data. J. Gerontol. A Biol. Sci. Med. Sci..

[27] Bouscaren N., Dartois L., Boutron-Ruault M.C., Vercambre M.N. (2018). How do self and proxy dependency evaluations agree? Results from a large cohort of older women. Age Ageing.

[28] Zhang T., Xu Y., Ren J., Sun L., Liu C. (2017). Inequality in the distribution of health resources and health services in China: Hospitals versus primary care institutions. Int. J. Equity Health.

[29] Chokkanathan S., Mohanty J. (2017). Health, family strains, dependency, and life satisfaction of older adults. Arch. Gerontol. Geriatr..

[30] Yeung G.T.Y., Fung H.H. (2007). Social support and life satisfaction among Hong Kong Chinese older adults: Family first?. Eur. J. Ageing.

[31] Matthews F.E., Bennett H., Wittenberg R., Jagger C., Dening T., Brayne C., Cognitive Function, Ageing Studies (CFAS) Collaboration (2016). Who Lives Where and Does It Matter? Changes in the Health Profiles of Older People Living in Long Term Care and the Community over Two Decades in a High Income Country. PLoS ONE.

[32] Taube E., Jakobsson U., Midlöv P., Kristensson J. (2016). Being in a Bubble: The experience of loneliness among frail older people. J. Adv. Nurs..

[33] Svanström R., Sundler A.J., Berglund M., Westin L. (2013). Suffering caused by care-elderly patients’ experiences in community care. Int. J. Qual. Stud. Health Well-Being.

[34] Goodman C., Dening T., Gordon A.L., Davies S.L., Meyer J., Martin F.C., Gladman J.R., Bowman C., Victor C., Handley M. (2016). Effective health care for older people living and dying in care homes: A realist review. BMC Health Serv. Res..

[35] Rivero-Jiménez B., Conde-Caballero D., Mariano-Juárez L. (2020). Health and Nutritional Beliefs and Practices among Rural Elderly Population: An Ethnographic Study in Western Spain. Int. J. Environ. Res. Public Health.

[36] Alcañiz M., Brugulat P., Guillén M., Medina-Bustos A., Mompart-Penina A., Solé-Auró A. (2015). Risk of dependence associated with health, social support, and lifestyle. Rev. Saúde Pública.

[37] Darbà J., Kaskens L. (2015). Relationship between patient dependence and direct medical-, social-, indirect-, and informal-care costs in Spain. Clinicoecon. Outcomes Res..

[38] Tabali M., Ostermann T., Jeschke E., Dassen T., Heinze C. (2013). Does the care dependency of nursing home residents influence their health-related quality of life?-A cross-sectional study. Health Qual. Life Outcomes.

[39] Barbareschi G., Sanderman R., Kempen G.I., Ranchor A.V. (2009). Socioeconomic status and the course of quality of life in older patients with coronary heart disease. Int. J. Behav. Med..

[40] Xu X., Zhou L., Antwi H.A., Chen X. (2018). Evaluation of health resource utilization efficiency in community health centers of Jiangsu Province, China. Hum. Resour. Health.

[41] Lum T.Y.S., Lou V.W.Q., Chen Y.Y., Wong G.H.Y., Luo H., Tong T.L.W. (2016). Neighborhood Support and Aging-in-Place Preference Among Low-Income Elderly Chinese City-Dwellers. J. Gerontol. B Psychol. Sci. Soc. Sci..

[42] Ishizaki T. (1992). Factors facilitating the return of the elderly to their homes after discharge from a geriatric intermediate care facility. Jpn. J. Public Health [Nihon Koshu Eisei Zasshi].

